# (−)-Epicatechin Inhibits Metastatic-Associated Proliferation, Migration, and Invasion of Murine Breast Cancer Cells In Vitro

**DOI:** 10.3390/molecules28176229

**Published:** 2023-08-24

**Authors:** Javier Pérez-Durán, Aglaé Luna, Andrés Portilla, Pamela Martínez, Guillermo Ceballos, Miguel Ángel Ortíz-Flores, Juan Mario Solis-Paredes, Nayelli Nájera

**Affiliations:** 1 Departamento de Investigación en Salud Reproductiva y Perinatal, Instituto Nacional de Perinatología Isidro Espinosa de los Reyes, Mexico City 11000, Mexico; djavier40@gmail.com (J.P.-D.); aglae.lunaflores@gmail.com (A.L.); juan.mario.sp@gmail.com (J.M.S.-P.); 2 Escuela Superior de Medicina, Instituto Politécnico Nacional, Mexico City 11340, Mexico; andresmeaney@gmail.com (A.P.); pam901111@gmail.com (P.M.); gceballosr@ipn.mx (G.C.); angelito_coa@hotmail.com (M.Á.O.-F.)

**Keywords:** (−)-epicatechin, breast cancer, metastasis

## Abstract

Breast cancer, due to its high incidence and mortality, is a public health problem worldwide. Current chemotherapy uses non-specific cytotoxic drugs, which inhibit tumor growth but cause significant adverse effects. (−)-Epicatechin (EC) is part of a large family of biomolecules called flavonoids. It is widely distributed in the plant kingdom; it can be found in green tea, grapes, and cocoa. Several studies in animals and humans have shown that EC induces beneficial effects in the skeletal muscle and the cardiovascular system, reducing risk factors such as arterial hypertension, endothelial dysfunction, damage to skeletal muscle structure, and mitochondrial malfunction by promoting mitochondrial biogenesis, with no adverse effects reported. Recently, we reported that EC had an antitumor effect in a murine triple-negative mammary gland tumor model, decreasing tumoral size and volume and increasing survival by 44%. This work aimed to characterize the effects of flavanol EC on proliferation, migration, and metastasis markers of triple-negative murine breast (4T1) cancer cells in culture. We found proliferation diminished and Bax/Bcl2 ratio increased. When the migration of culture cells was evaluated, we observed a significant reduction in migration. Also, the relative expression of the genes associated with metastasis, *Cdh1*, *Mtss1*, *Pten*, *Bmrs*, *Fat1*, and *Smad4*, was increased. In conclusion, these results contribute to understanding molecular mechanisms activated by EC that can inhibit metastatic-associated proliferation, migration, and invasion of murine breast cancer cells.

## 1. Introduction

Breast cancer, due to its high incidence and mortality, is a public health problem worldwide. It is the most common malignant disease and among the leading causes of cancer-related death in women [1,2].

Triple-negative breast cancer subtype is negative for estrogen receptor (ER), progesterone receptor (PR), and human epidermal growth factor receptor 2 (HER2). Expression is more prevalent among young women and accounts for 10–20% of newly diagnosed breast cancer cases. It is the most aggressive type of breast cancer and has a poorer prognosis compared with other breast cancer subtypes.

The success in the treatment of breast cancer depends, to a large extent, on early detection (TMN0-I) [3]. However, in developing countries, many patients are diagnosed in advanced stages, with regional (TNMII-III) or distant (TNM-IV) metastases, drastically decreasing the 5-year survival rate [4].

Metastasis (met), the process by which cancer cells migrate to adjacent tissue or spread to other parts of the body, involves a series of characteristics, such as dissociation from the primary tumor, migration, invasion, intravasation, and extravasation [5].

Current chemotherapy uses non-specific cytotoxic drugs, such as Adriamycin and cyclophosphamide, which inhibit tumor growth but cause significant adverse effects. In cost–benefit terms, the goal of increasing 5-year survival is achieved, but not necessarily with a good quality of life [6].

In the search for molecules that could increase treatment efficacy and possibly reduce adverse effects, multiple alternatives have been explored, including bioactive phytochemicals, which represent a possible low-cost, low-toxicity pharmacological approach that could impact patients and their quality of life as well as the health sector economy.

(−)-Epicatechin (EC) is part of a large family of biomolecules called flavonoids. It is widely distributed in nature; it can be found in green tea, grapes, and cocoa. Several studies in animals and humans have shown that EC induces beneficial effects on the skeletal muscle and the cardiovascular system, reducing risk factors such as arterial hypertension, endothelial dysfunction, damage to skeletal muscle structure, and mitochondrial malfunction by promoting mitochondrial biogenesis, with no adverse effects reported [7,8,9,10,11,12].

EC-induced effects include antioxidant, anti-inflammatory, cardio- and chemoprotective processes. In addition, antiproliferative effects have been described in different types of cancer, including breast, prostate, and lung [13,14,15].

Recently, we reported that EC induced an antitumor effect in a murine model of breast cancer, decreasing tumoral size and volume and increasing the survival of mice by 44% compared to the untreated group and 14% compared with a doxorubicin-treated group [13]. This increase in survival allows us to hypothesize that the effects might be related to a reduction in metastatic processes in the tumor. This work aimed to characterize the effects of flavanol (–)–epicatechin on proliferation, migration, and metastasis markers of triple-negative murine breast (4T1) cancer cells in culture.

## 2. Results

### 2.1. Cell Survival

**Figure 1:**
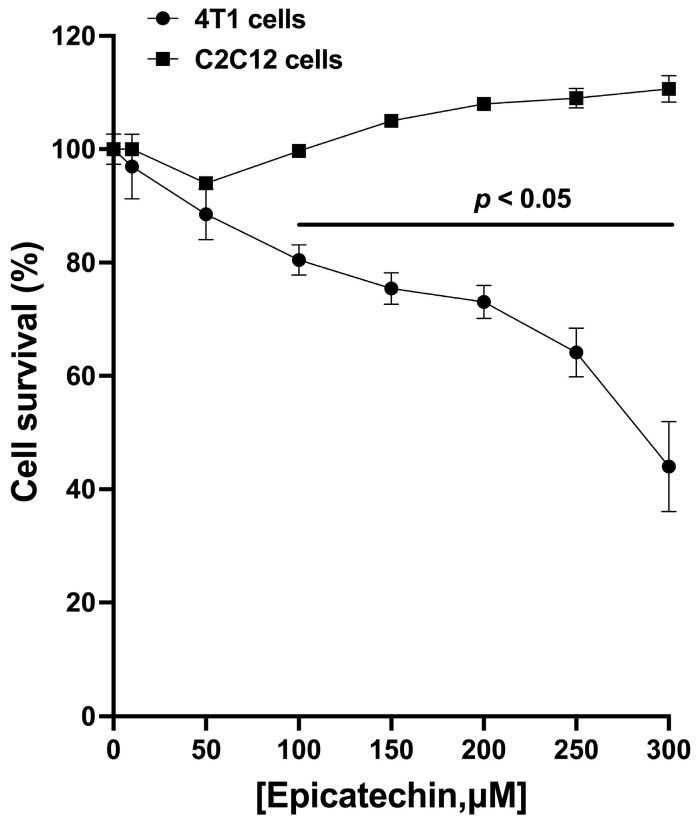
Tumor cell survival inhibition curve (4T1, circles) at different (−)-epicatechin concentrations. The most significant effect was achieved with 300 μM EC. The presence of EC in the culture of non-tumor cells (myoblasts C2C12, squares) did not cause a decrease in cell survival. Results are represented as the mean ± standard error of the mean (SEM). Experiments were performed in triplicate in at least two independent assays.

In 4T1 cells in culture, EC reduces cell survival in a concentration-dependent manner. A statistically significant reduction in survival was observed from 50 μM, reaching the greatest effect at 300 μM. The vehicle used (water) did not induce a decrease in survival (Figure 1). In an attempt to evaluate cytotoxicity in non-tumoral cells, the non-tumor cell line C2C12 (mouse myoblasts) was used to show the specificity of EC action. (−)-Epicatechin did not reduce myoblast survival at any of the concentrations tested (Figure 1).

### 2.2. Cell Death Bax/Bcl2 Ratio

**Figure 2:**
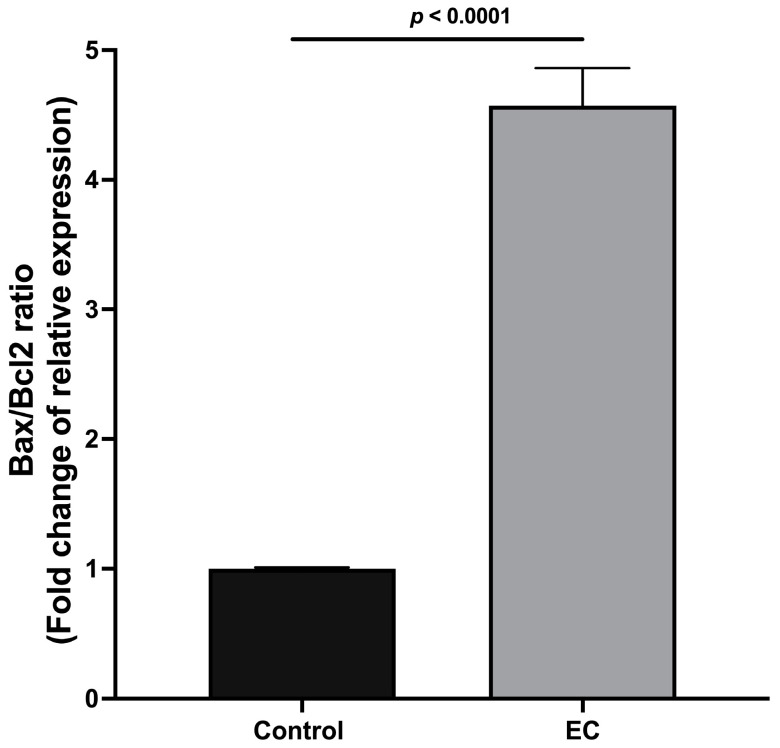
Analysis of the Bax/Bcl2 ratio without and with 300 μM EC. Results are normalized to controls and represented as the mean ± standard error of the mean (SEM). Experiments were performed in triplicate in at least two independent assays.

The Bax/Bcl2 ratio was calculated to identify whether the EC effects lead toward cell survival or death. The results show that the Bax/Bcl2 ratio increased significantly when the cells were incubated with EC (300 μM), decreasing cell viability (Figure 2).

### 2.3. Cell Migration Wound Healing

**Figure 3:**
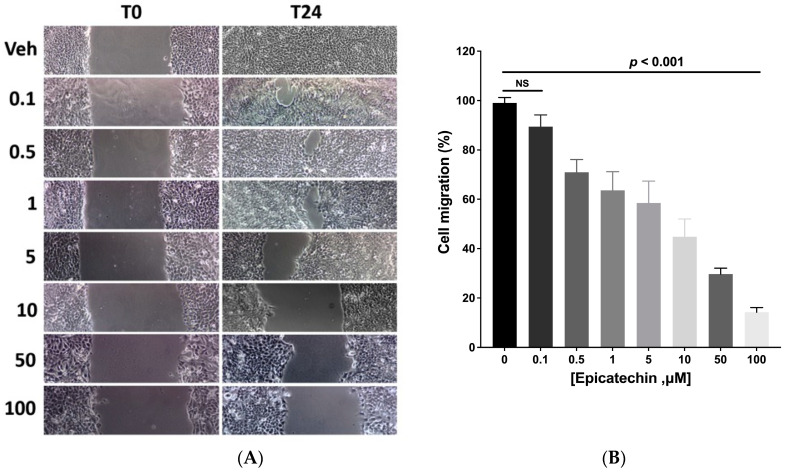
Wound healing test. (**A**). Representative micrographs of the wound were made in the cultures and 24 h later at different EC (μM) concentrations. (**B**) Wound closing percentage under EC treatment; closure under the vehicle was 100%, and EC induced a concentration-dependent decrease in closure (linear coef. = 0.9838). Results are represented as the mean ± standard error of the mean (SEM). Experiments were performed in triplicate in at least two independent assays.

The migration capacity of the tumor cells was evaluated using a wound closure assay produced in a monolayer culture of 4T1 cells. The wound area of cells with different concentrations of EC was evaluated. A concentration-dependent effect was observed; as the (−)-epicatechin concentration increased, the cells had reduced ability to migrate and close the wound space (Figure 3A). Twenty-four hours after the wound, the control cells migrated and closed the space. In contrast, as the EC concentration increased, the wound remained open. The maximum effect was obtained with the concentration of 100 μM, where the wound remained open to 90% (Figure 3B). EC induced a concentration-dependent effect with a linear behavior (Figure 3B, R2 = 0.9838).

### 2.4. Cell Invasion Assay

**Figure 4:**
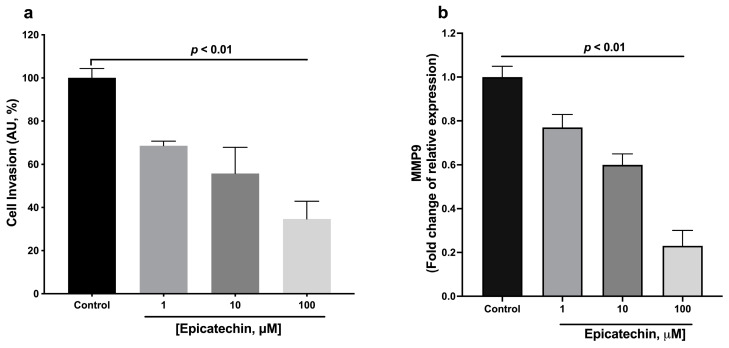
Evaluation of the invasive capacity of 4T1 cells. (**a**) Evaluation with Boyden camera. (**b**) Relative expression of metalloproteinase 9 (Mmp9). Results are represented as the mean ± standard error of the mean (SEM). Experiments were performed in triplicate in at least two independent assays.

In this assay, we only explore the effects 1, 10, and 100 μM of EC. It was observed that the invasive capacity of the 4T1 cells decreased significantly with EC treatments as compared to the control group (Figure 4a).

As part of the assessment of invasiveness, the relative expression of *Mmp9* metalloproteinase was quantified. EC decreased the expression of *Mmp9* in a concentration-dependent manner (Figure 4b).

### 2.5. Expression of Genes Related to the Metastasis Process

**Figure 5:**
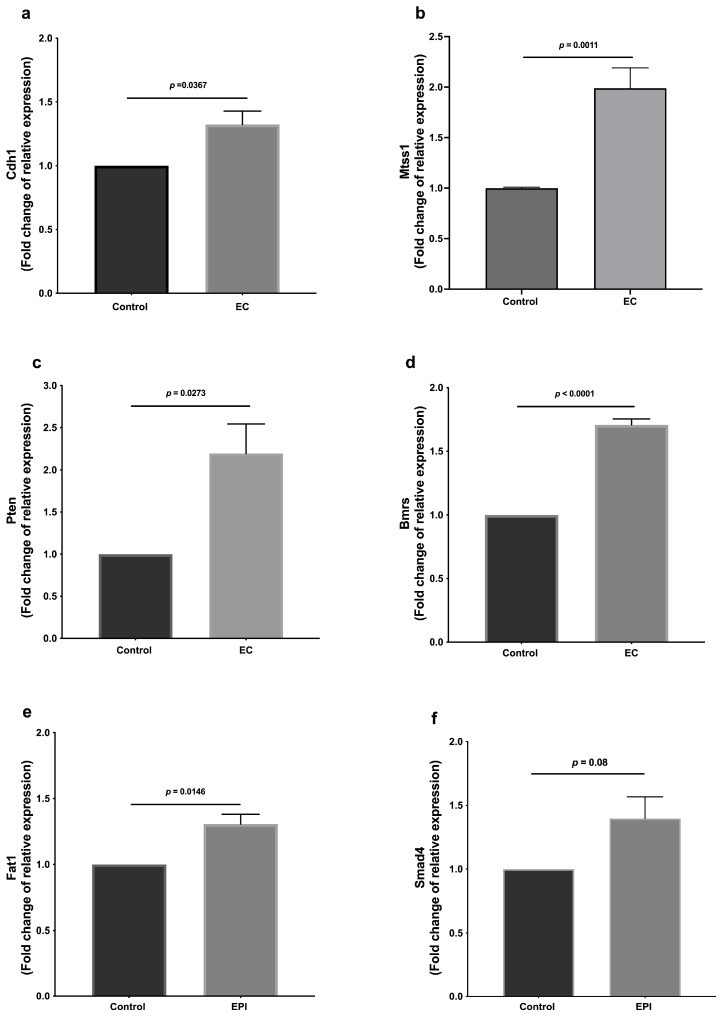
Relative expression of genes associated with metastasis: (**a**) *Cdh1*, (**b**) *Mtss1*, (**c**) *Pten*, (**d**) *Bmrs*, (**e**) *Fat1*, and (**f**) *Smad4*. Results are normalized to controls and represented as the mean ± standard error of the mean (SEM). Experiments were performed in triplicate in at least two independent assays.

**Figure 6:**
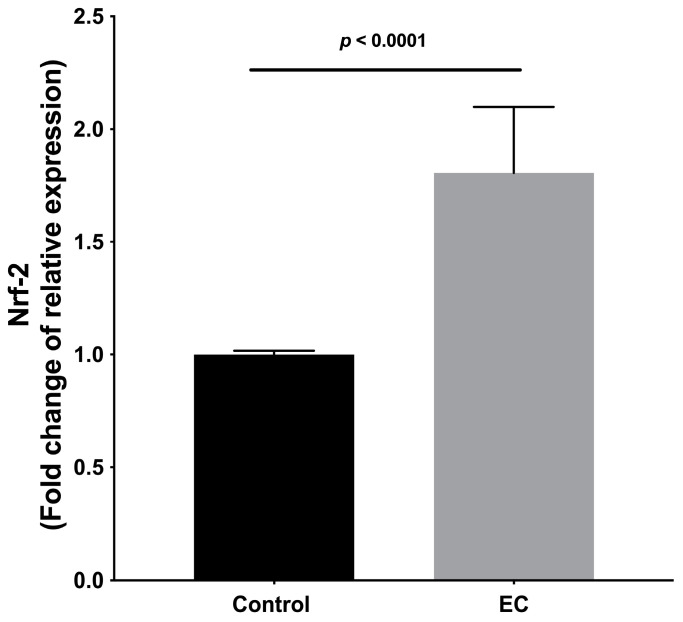
Relative expression of *Nrf2*. Results are normalized to controls and represented as the mean ± standard error of the mean (SEM). Experiments were performed in triplicate in at least two independent assays.

Gene expression was evaluated in treated and untreated tumor cells; it was observed that the treatments with EC (300 μM) induced higher expression of the anti-proliferative genes *Cdh1*, *Mtss1*, *Pten*, *Bmrs*, *Fat1*, *Smad4* (Figure 5), and *Nrf2* (Figure 6).

## 3. Discussion

**Figure 7:**
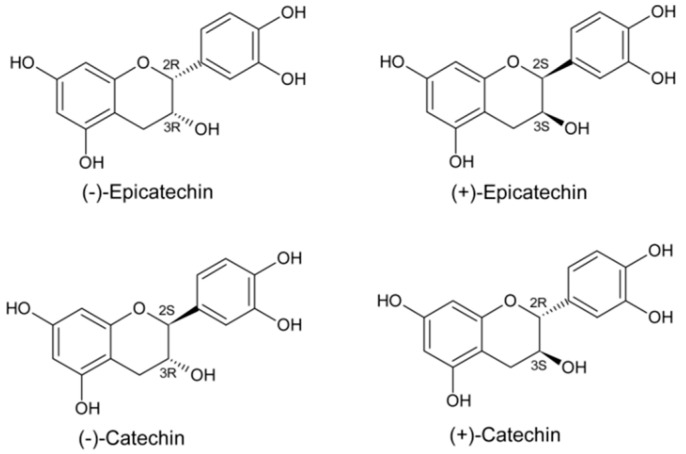
Structural characteristics of flavanols or flavan-3-ols. Catechins comprise a subfamily of flavonoids, which structurally do not possess a double bond between the C2 and C3 carbons or a carbonyl group on the C ring. Due to these characteristics and hydroxylation on the C3, the configuration of four diastereoisomers results: the trans (2S, 3R) and (2R, 3S) isomers, called catechins, and the cis (2S, 3S) and (2R, 3R) isomers, called epicatechins.

(−)-Epicatechin is part of a family of secondary metabolites in several vegetal products, known as catechins. It is the most abundant flavanol in cacao. There are four isomers (diastereoisomers, see Figure 7) in nature.

The (−)-epicatechin-induced effects seem specific, since isomers like catechin, of the same chemical characteristics and differing only in the substituents’ spatial orientation, induce no or limited effects in several setups [16,17]. We reported that (−)-epicatechin is superior to (+)-catechin in activating the upstream signaling pathways associated with mitochondrial biogenesis. The stereoisomer (+)-epicatechin is substantially more active than (−)-epicatechin in stimulating the protein levels of electron transport chain complexes II and IV, as well as mitochondria-related endpoints in cultured cells and treated mice [17]. These findings suggest highly selective molecular interactions with possible receptors and these flavanols.

In a murine model of triple-negative mammary gland cancer, we showed that EC reduces the tumor volume by up to 74% compared to the control group. Interestingly, the effect was similar to that generated by Doxycycline [13].

The similarity between these two molecules’ induced effects is relevant considering the adverse effects that conventional chemotherapy induces, including cardiotoxicity, which limits its use or induces a decrease in quality of life. These results have great potential for application, since EC is considered a GRAS (generally recognized as safe) substance by the FDA, and no adverse effects are reported in the literature.

In searching for the molecular mechanisms through which EC acts, we analyzed the AMPK and Akt/mTOR pathways as participants in regulating cell proliferation. The reported results showed that the activation of AMPK increased. In this sense, the homeostasis and adaptation to metabolic stress in cancer cells are mainly due to the integral response exerted by activating the energy sensor AMPK. Results also showed that Akt phosphorylation was diminished. It has been reported that the constitutive phosphorylation of Akt in cancer cells activates different targets for tumor development as a pathway to chemoresistance in cancer, and the PI3K/Akt/mTOR pathway inhibition has been associated with high survival rates in different types of cancer.

The present study is a follow-up of the murine model. It has been reported that 4T1 cells have a high capability of metastasizing, and the increase in survival may also be related to an inhibition of the metastatic process. We believed that these phenomena could be more easily evaluated in vitro. Consequently, we evaluated the effects of (−)-epicatechin in murine breast cancer (4T1) cell proliferation, migration, and invasive capacity in vitro. The results showed that EC is a cytotoxic molecule that specifically affects cancer cells, since C2C12 cells were not affected similarly. Treatment with the flavanol increased the Bax/Bcl2 ratio, suggesting increased sensibility for apoptosis. EC also induced a concentration-dependent decrease in 4T1 cell migration capability, with decreases in cell invasion. Interestingly, EC also induced a decrease in metastatic molecular markers. These results altogether demonstrate the EC effect in decreasing the 4T1 cells’ metastatic potential.

There is evidence of EC antitumor effect, although the molecular mechanisms through which it exerts this effect have not yet been well characterized.

In murine breast cancer, we previously demonstrated that EC’s antiproliferative effect is related to regulating AMPK and Akt/mTOR signaling pathways [13]. This antiproliferative effect was also observed in A549 lung cancer cells in culture [18].

The effect of EC is reflected in not only the decrease in the tumor size (antiproliferative effect) but also in the induced increase in the survival of the animals by up to 44% [13]. However, the increase in survival may not depend solely on the control of tumor size but also on other variables, such as the reduction in the metastatic capacity of tumor cells. In the present study, we explored the effect of EC on breast cancer cell proliferation and migration to validate the possibility of EC being an antimetastatic agent in this triple-negative breast cancer cell line model. These results and its effects in vivo as antitumoral allow us to propose it as a viable agent to be used with lower doses of chemotherapeutics. However, more work is necessary to fully characterize EC’s effects.

The reported results using the MTT assay show cell viability decreases in a concentration-dependent manner in tumor cells (4T1) but not in non-tumor cells (myoblasts). An indicator of the pathway that could be activated is the Bax/Bcl2 ratio. We found that exposure of tumor cells to EC significantly increases the Bax/Bcl2 ratio. It has been proposed that this ratio functions as a modulator, determining susceptibility to apoptosis, such that a decreased ratio is associated with tumor cells resistant to apoptosis (malignancy). In contrast, an increased ratio of Bax/Bcl is associated with cells sensitive to apoptosis [19,20].

It has also been discussed that the dysregulation of the Bax/Bcl2 ratio could be related to other malignant characteristics, such as cell invasion and adhesion [21,22]. Thus, in accordance with the above, and as per the Warburg effect, the search for drugs that regulate mitochondrial function remains current. (−)-Epicatechin is thus an ideal candidate molecule, since it promotes biogenesis and improves mitochondrial structure and function [22,23].

On the other hand, we observed that EC reduced cell migration and closed the wound caused by the 4T1 cell monolayer. EC presence also reduced the expression of Mmp9, an enzyme necessary to degrade extracellular matrix components such as type IV and V collagen. Also, there was a significant reduction in the number of cells that crossed the protein components of Boyden’s chambers, indicating that EC promotes a decrease in migration and the invasive capacity of 4T1 tumor cells.

Similarly, we also observed an increase in the expression of Cdh-1, a membrane protein involved in the cell–cell union that might be involved in the decrease of the epithelial-mesenchymal transition process; these intercellular junctions restrict cell movement and migration and inhibit proliferation, increasing apoptosis and decreasing the cells capable of migration and metastasis.

In this regard, cadherins are essential for cell adhesion and include FAT atypical cadherins (FAT), one of the most frequently mutated genes in human cancer. *Fat1* expression can inhibit cell proliferation, colony formation, and cell migration and invasion, whereas Fat1-knockout induces increases in migration and tumor invasion [24]. Remarkably, EC increased *Fat1* expression, showing a relevant effect of the flavanol reducing metastatic-associated pathways.

Other molecules were also affected by EC treatment. MTSS1 expression levels are inversely correlated with the clinical pathology and prognosis in triple-negative breast cancer (TNBC) tissues [25,26]. MTSS1 protein binds to actin and promotes cytoskeletal organization, which inhibits the tumor cell metastatic capacity by inhibiting epithelial-to-mesenchymal transition [26]. It also inhibits the cell–cell junctional disassembly induced by enhancing the strength of the cell–cell junction and accelerates the kinetics of the adherent junction assembly [26]. EC induced an increase in Mtss1 expression.

Our results also showed an increase (even when non-significant, *p* = 0.08) in SMAD4. SMAD4 is the mediator of the TGF-b pathway, playing a tumor-suppressive role, particularly at early stages, since it induces cell cycle arrest and apoptosis and correlates negatively with tumor metastasis. The decrease or deletion of SMAD4 promotes tumorigenesis, significantly when PTEN is decreased or deleted.

PTEN (Phosphatase and Tensin homolog) is a tumor suppressor gene related to SMAD4 function; the loss of its expression/function may be related to cancer aggressiveness. Recently, it has been reported that inhibition of PTEN (using VO-OHpic) in 4T1 cells is associated with increased Akt phosphorylation and the PI3K key signaling pathways regulating cell proliferation and death, suggesting that PTEN loss or deletion increase proliferation, invasion, and metastasis in 4T1 cells [27]. EC increased the *Pten* expression levels.

In summary, the decrease or loss of CDH1 (E-cadherin gene), SMAD4, PTEN, and FAT1 expression leads to an aggressive tumor with an increased invasive phenotype and increased metastatic potential. Interestingly, the results reported in this work show that EC increases the expression of *Cdh1*, *Smad*, *Pten*, and *Fat1*, suggesting that the flavanol can decrease the metastatic potential in a coordinated manner.

On the other hand, it has been reported that breast cancer metastasis suppressor 1 (BRMS1) inhibits metastasis in several cancer types, such as ovarian cancer, bladder cancer, melanoma, and breast cancer [28,29,30]. In metastasis mouse models, BRMS1 has a high capacity to inhibit metastatic processes. It has also been demonstrated that loss of or decrease in BRMS1 expression is present in several human tumors, such as breast cancer. Our results shown that EC increases the expression of Brms1, suggesting that this flavanol can interfere with this pathway and decrease the metastatic potential of 4T1 cells.

On the other hand, EC increased the expression of *Nrf2*. In this regard, it has been reported that Nfr2 has protective roles in suppressing oxidative or electrophilic stress and inhibiting carcinogenesis [31].

Stimulation of Nrf2 has been proposed as a cancer prevention method [32,33]. Nrf2 activators, such as curcumin and resveratrol, have been shown to induce cryoprotection. However, it has also been proposed that null (Nfe2l2−/−) mice are more susceptible to chemical- and radiation-induced tumorigenesis, and NRF2 activators were reported to reduce the burdens of breast cancer [34].

These dual roles as pro-oncogenic and anti-oncogenic factors depend on metabolic adaptation, cell proliferation, and the induction level of Nrf2 [35].

From the metabolic point of view, NRF2 deficiency results in a decreased efficiency of oxidative phosphorylation, whereas NRF2 activation has the opposite effect [36]. ATP levels are significantly higher in cells with constitutive upregulation of Nrf2 and lower when Nrf2 is knocked down or disrupted [36]. Interestingly, due to the Warburg effect, cancer cells use glycolysis to produce anabolic precursors to support rapid tumor growth, resulting in a much less efficient production of ATP [37].

Activators of NFR2 cause an increase in mitochondrial biogenesis (mitochondrial mass) and induction of transcriptional coactivators, such as peroxisome proliferator-activated receptor coactivators (PGC)1α and 1β [38]. EC treatment induces similar stimuli and increases mitochondrial biogenesis through NFR2 and (PGC)1α and 1β [39]. We hypothesized that this phenomenon could be related, in cancer cells, to a switch in the metabolism, making the tumoral cells more mitochondrial-dependent. However, more work is necessary to characterize these processes.

In summary, the data reported here suggest that treatment with EC induces a decrease in the metastatic process (decrease in the molecular markers associated with metastasis) in a murine model (4T1 cells) of triple-negative breast cancer.

Altogether, the results suggest that (−)-epicatechin can be seen as an alternative therapeutic approach that may serve as an adjuvant to improve therapeutical efficacy.

## 4. Materials and Methods

### 4.1. 4T1 Cell Culture

4T1 cells (ATCC CRL-2539) were cultured under standard conditions using RPMI-1640 medium (Biowest L0498-500), with 10% fetal bovine serum and 1% antibiotic, and with controlled temperature (37 °C) and an atmosphere of 5% CO_2_.

Cells were grown in a monolayer to 80% confluence. The following tests were carried out.

### 4.2. Cytotoxicity Test in 4T1 Cells in Culture

A total of 5 × 10^4^ 4T1 cells were seeded in 96-well plates. The cells were treated with different concentrations of EC (0, 1, 3, 10, 30, 100, and 300 μM) for 5 days under standard culture conditions, changing the culture medium every other day. After the incubation period, cells were washed with HBSS (3×) and incubated with 0.1 mg/mL MTT (3-(4,5-dimethylthiazol-2-yl)-2,5-diphenyltetrazolium bromide; Sigma-Aldrich M2003) for 15 min at 37%. The wells were washed. The formazan crystals formed were dissolved with 100 μL of isopropanol. The absorbance of the solution was measured at 540 nm. Viability was expressed as a percentage compared with the control group (vehicle).

$$\%\;cell\;viability=\frac{Average\;absorbance\;experimental\;group\;}{Average\;absorbance\;control\;group}\;\times \;100$$

### 4.3. Cytotoxicity Test in C2C12 Cells in Culture

To explore the EC effects on non-cancer cells, we analyzed the viability of C2C12 myoblasts. These cells are of skeletal muscle origin and do not shear the cancer-related characteristics of the 4T1 cell type. Cytotoxicity was evaluated using MTT assay (see above).

### 4.4. Wound Healing

A total of 5 × 10^4^ 4T1 cells were seeded in 24-well plates. After 24 h, a wound was made in the middle of the well using a sterile 200 µL micropipette tip. The culture medium was removed, and cells were washed with HBSS to leave a cell-free area. Cell wells were randomly treated with different EC concentrations (0, 0.5, 0.1, 1, 5, 10, 50, and 100 µM). Photographs (10×) of the wound at time zero (T0) were taken, and the plate coordinates were recorded. The cells were incubated for 24 h with the different EC concentrations. Photographs were taken according to the registered coordinates. Wound measurements were calculated with ImageJ software.

$$\%\;relative\;area=\frac{Average\;area\;experimental\;group\;}{Average\;area\;control\;group}\times 100$$

### 4.5. Cell Invasion Assay

The Cell Invasion Assay Kit (ECM550, Chemicon International Inc, Temecula, CA, USA) was used, and the assay was performed according to the supplier’s instructions. Briefly: chambers were rehydrated with serum-free RPMI culture medium for 2 h. Subsequently, a new suspension of 3 × 10^4^ 4T1 cells in serum-free RPMI medium was placed in the chamber. The chambers were placed in a well containing 500 μL of medium supplemented with 10% fetal bovine serum, which functioned as a chemoattractant. The plates were incubated under standard culture conditions. After 24 h, the non-invasive cells in the chamber’s inner part were removed with a cotton-tipped swab. The invading cells on the underside of the chamber were stained with a staining solution. Water washes were performed to remove excess. The inserts were immersed in wells with 100 μL of 10% acetic acid for their colorimetric reading of OD at 560 nm.

### 4.6. Analysis of Genes Related to Proliferation and Metastasis by Q-PCR

A total of 6 × 10^5^ 4T1 cells were seeded in Petri dishes and treated with EC 300 μM for 5 days; total RNA was extracted according to the supplier’s instructions for the Direct-zol RNA Miniprep Plus kit (R2051, Zymo Research, Irvine, CA, USA). Synthesis of cDNA was performed on 2 µg of total RNA using the QuantiTect Reverse Transcription kit and RT-qPCR (ID: 20531, Qiagen, Valencia, CA, USA), following the indications described by the supplier. Relative expression of the selected genes was determined with Biorad’s SSofast Eva Green kit on a CFX96 Touch Real-Time PCR Detection System (Biorad, Hercules, CA, USA). Oligonucleotides used are presented in Table 1.

The PCR conditions were 95 °C for 20 s, followed by 40 cycles of 15 s at 95 °C and 30 s at 60 °C. Assays were performed in triplicate, and actin mRNA was amplificated as a constitutive gene. Results were normalized to the 4T1 control by the comparative CT method (∆∆CT).

### 4.7. Statistical Analysis

All assays were performed in triplicate in at least two independent assays. Results are represented as the mean ± standard error of the mean. Statistical analysis was performed with GraphPad Prism version 9.5.1 software (San Diego, CA, USA). Multiple comparisons were performed with one-way analysis of variance (ANOVA) with Tukey’s post hoc test. In the case of the comparison of two groups, the t-student test was used. A value of *p* < 0.05 was considered statistically significant.

## 5. Conclusions

In conclusion, these results contribute to understanding the molecular mechanisms activated by EC that can inhibit metastatic-associated proliferation, migration, and invasion of murine breast cancer cells; the proposal to use EC as an adjuvant agent in breast cancer is supported.

## Figures and Tables

**Table 1:** Oligonucleotides used.

Gene	Forward Primer (5′ to 3′)	Reverse Primer (5′to 3′)	Reference
*Bax*	AGGATGCGTCCACCAAGAAGCT	TCCGTGTCCACGTCAGCAATCA	[40]
*Bcl2*	CCTGTGGATGACTGAGTACCTG	AGCCAGGAGAAATCAAACAGAGG	[40]
*Mmp9*	TGTTCCCGTTCATCTTTGAG	ATCCTGGTCATAGTTGGCTGT	[41]
*Cdh1*	CTCCAGTCATAGGGAGCTGTC	TCTTCTGAGACCTGGGTACAC	ID: 118129809c3
*Mtss1*	ATGGAGGCTGTGATCGAGAAG	CCAAACTGGATAGCTCCCCT	ID: 226052051c1
*Pten*	TGGATTCGACTTAGACTTGACCT	GCGGTGTCATAATGTCTCTCAG	ID: 6679523a1
*Brms*	GGTGGACTACGCGGAGAAC	CACCTGACTCAACCGCTCTTT	ID: 27754014a1
*Fat1*	CTACGGAGGAACGTGCATGG	ATCTTTGCAGTACGGACTAAGC	ID: 27697111a1
*Smad4*	ACACCAACAAGTAACGATGCC	GCAAAGGTTTCACTTTCCCCA	ID: 28201436a1
*Nrf2*	TAGATGACCATGAGTCGCTTGC	GCCAAACTTGCTCCATGTCC	[42]

## Data Availability

Not applicable.

## Sample Availability

Samples of the compounds are available from the authors.
